# Clinician Burnout and Effectiveness of Guideline-Recommended Psychotherapies

**DOI:** 10.1001/jamanetworkopen.2024.6858

**Published:** 2024-04-17

**Authors:** Nina A. Sayer, Adam Kaplan, David B. Nelson, Shannon Wiltsey Stirman, Craig S. Rosen

**Affiliations:** 1 Center for Care Delivery and Outcomes Research, Minneapolis VA Health Care System, Minneapolis, Minnesota; 2 Department of Medicine, University of Minnesota, Minneapolis; 3 Department of Psychiatry and Behavioral Sciences, University of Minnesota, Minneapolis; 4 National Center for Posttraumatic Stress Disorder, Dissemination and Training Division at the Veterans Affairs Palo Alto Health Care System, Palo Alto, California; 5 Department of Psychiatry and Behavioral Sciences, Stanford University School of Medicine, Stanford, California

## Abstract

**Importance:**

Clinician burnout has been associated with clinician outcomes, but the association with patient outcomes remains unclear.

**Objective:**

To evaluate the association between clinician burnout and the outcomes of patients receiving of guideline-recommended trauma-focused psychotherapies for posttraumatic stress disorder (PTSD).

**Design, Setting, and Participants:**

This cohort study was set at the US Veterans Affairs Health Care System and included licensed therapists who provided trauma-focused psychotherapies and responded to an online survey between May 2 and October 8, 2019, and their patients who initiated a trauma-focused therapy during the following year. Patient data were collected through December 31, 2020. Data were analyzed from May to September 2023.

**Exposures:**

Therapists completing the survey reported burnout with a 5-point validated measure taken from the Physician Worklife Study. Burnout was defined as scores of 3 or more.

**Main Outcomes and Measures:**

The primary outcome was patients’ clinically meaningful improvement in PTSD symptoms according to the PTSD Checklist for the *Diagnostic and Statistical Manual of Mental Disorders* (*Fifth Edition*). Patient dropout, therapist adherence, and session spacing was assessed through electronic health records. Multivariable random-effects logistic regression examined the association of therapist burnout and clinically meaningful improvement, adjusted for case-mix.

**Results:**

In this study, 165 of 180 (91.7%) therapists (89 [53.9%] female) completed the burnout measure and provided trauma-focused psychotherapies to 1268 patients (961 [75.8%] male) with outcome data. Fifty-eight (35.2%) therapists endorsed burnout. One third of patients (431 [34.0%]) met criterion for clinically meaningful improvement. Clinically meaningful improvement in PTSD symptoms was experienced by 120 (28.3%) of the 424 patients seen by therapists who reported burnout and 311 (36.8%) of the 844 patients seen by therapists without burnout. Burnout was associated with lower odds of clinically meaningful improvement (adjusted odds ratio [OR],0.63; 95% CI, 0.48-0.85). The odds of clinically meaningful improvement were reduced for patients who dropped out (OR, 0.15; 95% CI, 0.11-0.20) and had greater session spacing (OR, 0.80; 95% CI, 0.70-0.92). Therapist adherence was not associated with therapy effectiveness. Adjusting for dropout or session spacing did not meaningfully alter the magnitude of the association between burnout and clinically meaningful improvement.

**Conclusions and Relevance:**

In this prospective cohort study, therapist burnout was associated with reduced effectiveness of trauma-focused psychotherapies. Studying when and how burnout affects patient outcomes may inform workplace interventions.

## Key Points

**Question:**

Is therapist burnout associated with reduced effectiveness of guideline-recommended psychotherapies for posttraumatic stress disorder (PTSD)?

**Findings:**

In this cohort study of 165 therapists and 1268 patients, therapist burnout was significantly associated with reduced effectiveness of guideline-recommended psychotherapies for PTSD. The proportion of patients who experienced clinically meaningful improvement in PTSD symptoms was 28.3% among therapists who reported burnout and 36.8% among therapists without burnout.

**Meaning:**

These findings suggest that interventions to reduce therapist burnout might also result in more patients experiencing clinically meaningful improvement in PTSD symptoms from evidence-based psychotherapies.

## Teaser

This cohort study evaluates the association of therapist burnout with the effectiveness of guideline-recommended trauma-focused psychotherapies for posttraumatic stress disorder among patients.

## Introduction

Burnout among US health care professionals is an epidemic that preceded and has been exacerbated by the COVID-19 pandemic.^1,2^ The toll of burnout includes physical and mental health symptoms as well as the intention to leave the profession, potentially worsening workforce shortages.^3,4^ Burnout may also affect the quality of care that clinicians who remain in the workforce provide. Clinicians experiencing burnout self-report lower quality of care, poorer communication with patients, and more medical errors.^5,6,7,8^ However, burnout is not consistently associated with evidence of poorer quality of care or worse outcomes.^5,7,9,10^ Health systems need information about whether reducing burnout may be important for treatment effectiveness.

The association of burnout on patient care may be particularly evident for interventions that require a high level of clinician empathy and interpersonal engagement, such as psychotherapy.^11^ Yet there has been surprisingly little research on patient outcomes associated with burnout among mental health professionals.^12^ One cross-sectional study^13^ found that therapists’ self-reported burnout was associated with poorer patient-reported outcomes from psychological interventions of different intensities for depression or anxiety.

We examined treatment outcome associated with burnout among licensed mental health professionals providing trauma-focused psychotherapies (TFPs) for posttraumatic stress disorder (PTSD) within the US Veterans Health Administration (VHA). All clinical practice guidelines for PTSD recommend TFPs as first line treatments for PTSD.^14^ VHA has widely implemented 2 TFPs—cognitive processing therapy and prolonged exposure—along with electronic health care record templates for tracking their delivery.^15,16^ Cognitive processing therapy and prolonged exposure are comparably effective manualized psychotherapies that involve 8 to 15 structured sessions delivered once or twice a week.^17,18,19,20^ Our primary aim was to evaluate the association between burnout and TFP effectiveness. We hypothesized reduced odds of clinically meaningful improvement among patients of therapists experiencing burnout. Our secondary aim was to explain how burnout may affect outcomes if our first hypothesis was supported. This included an examination of whether patient dropout and measures of TFP implementation quality would mediate the association between therapist burnout and patient improvement. Patient dropout before receiving an adequate dose is common for TFPs and associated with poorer outcomes.^21,22^ Alternatively, burnout might be associated with reduced effectiveness if therapists experiencing burnout diverge from the treatment protocols and deliver lower quality psychotherapy.

## Methods

This prospective cohort study was a secondary analysis of a study evaluating sources of variation between therapists in TFP outcomes.^23^ The Minneapolis VA Health Care System institutional review board (IRB) approved this research. We followed the Strengthening the Reporting of Observational Studies in Epidemiology (STROBE) reporting guideline.^24^

### Participants

The study population was licensed mental health professionals who provided TFPs and responded to an online survey between May 2 and October 8, 2019, and their patients who initiated a TFP over the following year. Allowing time for treatment completion, we included patient-level data through December 31, 2020.

To obtain a representative sample of therapists, we used administrative data to stratify the population of licensed mental health professionals who provided individual TFPs to at least 3 patients in the year before the start of the study into 12 strata based on the type of TFP provided (cognitive processing therapy, prolonged exposure, both) and census region (Midwest, Northeast, South, West). We randomly and proportionally selected therapists for recruitment within strata and used VHA email for therapist recruitment. Therapists completed informed consent online. Immediately after consenting, therapists completed a 15-minute online survey of their perceptions of their work environment.^25^ The parent study included 180 therapists from across the US who completed the survey and provided TFPs to at least 3 patients in the year following consent.^23^ The analyses presented here included therapists in the parent study who completed the burnout measure on the survey and had TFP patients with PTSD symptom scores recorded in the electronic health record, allowing for the calculation of treatment outcomes. We linked therapists who completed the burnout measure to their TFP patients using electronic health record templates and current procedure terminology codes for psychotherapy. A waiver of informed consent for patients was granted by the IRB. We used manual medical record review to verify that the included patients received at least 2 TFP sessions with the consented therapist.

### Measures

#### Burnout

The therapist survey included a nonproprietary, single-item burnout measure taken from the Physician Worklife Study that assesses participants’ self-defined level of burnout on a 5-category ordinal scale.^26^ This measure is mainly associated with the emotional exhaustion domain of burnout.^27^ Consistent with prior research,^7,28,29,30,31^ we dichotomized burnout with scores 2 or less (no symptoms of burnout) vs 3 or more (indications of burnout).

#### Outcome Measure

The TFP protocols specify assessment of PTSD symptoms using the PTSD Checklist for *Diagnostic and Statistical Manual of Mental Disorders* (Fifth Edition) (*DSM-5*) (PCL-5), a validated 20-item self-report measure to assess the *DSM-5* symptoms of PTSD.^32,33^ Higher scores indicate more severe PTSD symptoms. We used data extraction from the electronic health record supplemented with manual medical record review to identify total PCL-5 scores for each TFP session. When a PCL-5 score was unavailable for an initial session, we extracted PCL-5 scores from the preceding 2 weeks. If there were no initial or pretherapy PCL-5 scores, we used the earliest available score prior to the third session. To calculate symptom change, we used the PCL-5 score closest to the last session. Consistent with recent research in veterans,^34^ we used a reduction of 15 or more points on the PCL-5 to indicate clinically meaningful improvement.

#### Patient Dropout

We defined dropout as completing fewer than 8 CPT or PE sessions^35,36^ without meeting criterion for early completion. Early completers were patients who achieved a final PCL-5 score of 18 or less before 8 sessions^17^ or had an early completion templated medical record note, indicating symptom remission or treatment goals met. Early completers (57 patients) were grouped with completers for analysis.

#### Therapy Quality

The TFP protocols are highly specified, allowing for evaluation of therapy quality in terms of adherence and session spacing.^37^ To rate therapist adherence, we manually coded the documentation generated by TFP electronic health record templates, which include checklists for the essential elements of each therapy in each session. To obtain stable estimates of therapists’ adherence,^38^ we rated the first 7 sessions for 10 patients per therapist (or all patients if the therapist provided TFPs to fewer than 10 patients). Through this process we rated 6844 sessions for 1176 patients seen by the therapists in this patient sample. Therapists’ mean adherence scores (across sessions and patients) ranged from 46.5% to 100% and were negatively skewed (median [IQR], 89.2% [10.75%]).

Session spacing was defined as therapists’ mean days between TFP sessions averaged across their patients. According to the protocols, cognitive processing therapy is to be delivered in 60-minute and Prolonged Exposure in 90-minute weekly sessions, and efficacy trials for these TFPs often used a twice per week session structure.^19,20^ Mean session spacing for therapists ranged from 2.95 to 21.1 days and was roughly normally distributed (mean [SD], 11.3 [2.93] days).

### Therapist Characteristics

The survey included questions assessing clinic role, number of years treating veterans with PTSD, number of years since professional degree, race and ethnicity. Therapists indicated the race and ethnicity with which they identified according to investigator-defined categories (African American or Black, Asian American, Native American or Alaskan Native, Pacific Islander, White, and other). We extracted professional discipline, sex (gender was not available), clinic setting (PTSD specialty care, other mental health), and employment location from administrative data.

### Patient Characteristics

Patient characteristics, extracted from administrative databases, were age, military service era, sex (gender was inconsistently available in administrative records), race (Asian, Black, Hawaiian or Pacific Islander, Native American, White), ethnicity (Hispanic), disability status for military-related PTSD (PTSD service connection), past year psychiatric comorbidities, and past year medical comorbidities. We used past year medical comorbidities to compute Charlson Comorbidity Index scores.^39^ We manually extracted the following from clinical notes: education, employment, marital status, housing stability, index trauma for therapy and history of childhood trauma and multiple trauma. Because the COVID-19 pandemic began during data collection and may have impacted therapy delivery, we created a variable to classify each patient into 1 of 3 time periods depending on the date of therapy initiation. The prepandemic period included the 682 patients who began a TFP in 2019, the early pandemic period included the 256 patients who began a TFP in January or February 2020, the pandemic period included the 330 patients who began a TFP after March 2020.

### Statistical Analysis

Univariate descriptive analyses evaluated therapist and patient characteristics and the distribution of study measures. We used simple logistic regressions to identify variables independently associated with clinically meaningful improvement, using a Wald test *P* < .05, for inclusion as covariates in subsequent multivariable models. For categorical variables, a reference category was identified and when a nonreference category demonstrated no statistical difference from the reference category, that category was merged with the reference category. We fit a multivariable logistic regression model that included random effects for therapists and fixed effects for burnout, time period for treatment initiation, and the 5 qualifying patient case-mix variables, namely, standardized baseline PCL-5, age, being retired from employment, lacking stable housing, and depression diagnosis in the year before TFP initiation. Subsequently, we expanded this model to include the qualifying therapy delivery measures. We estimated model-based probabilities for the outcome, setting covariates at their mean for continuous variables or their mode for categorical variables. We used the delta method^40^ to compute 95% CIs for the differences between the estimated probabilities of clinically meaningful improvement given burnout and the other variables in the model.

We planned to evaluate whether the therapy delivery measures mediated the effect of burnout on clinically meaningful improvement (eAppendix in Supplement 1). However, mediation analyses were not implemented because these variables did not meet the eligibility criterion of significant association with the estimator.^41^

In sensitivity analyses, we evaluated whether the association between burnout and effectiveness changed when our outcome was defined as a PCL-5 reduction of 10 points or more (the commonly-used, consensus-based definition of improvement^33,42^) rather than 15 points or more (the empirically-based definition^34^ used in this study). Statistical analyses were performed with a 2-sided significance level of *P* < .05. Data were analyzed from May to September 2023 using R version 4.3.1. (R Project for Statistical Computing).

## Results

### Therapist Characteristics and Burnout Distribution

The therapist sample included 165 (91.7%) of the 180 therapists in the parent study. Most were psychologists (92 [55.8%]) or social workers (67 [40.6%]) and 89 (53.9%) were female (Table 1). Therapists worked in 115 different medical centers across the US.

**Table 1.** Characteristics of 165 Therapists Who Provided Trauma-Focused Psychotherapy to 1268 Patients

Characteristic	Therapists, No (%)
Sex^a^	
Female	89 (53.9)
Male	27 (16.4)
Missing	49 (29.7)
Race^b^	
Asian, Black, multiracial, other	16 (9.7)
White	145 (87.9)
Missing	4 (2.4)
Ethnicity^b^	
Hispanic	<7
Non-Hispanic	≥156 (94.5)
Missing	2 (1.2)
Discipline	
Psychologist	92 (55.8)
Social worker	67 (40.6)
Other	6 (3.6)
Missing	0
Years clinical experience since professional degree	
1-5	30 (18.2)
6-10	58 (35.2)
11-15	29 (17.6)
16-20	22 (13.3)
>20	25 (15.2)
Missing	1 (0.6)
Years treating veterans with PTSD	
<1	0
1-5	60 (36.4)
6-10	65 (39.4)
11-15	24 (14.5)
16-20	8 (4.8)
>20	7 (4.2)
Missing	1 (0.6)
Census region for workplace	
Midwest	43 (26.1)
Northeast	27 (16.4)
South	62 (37.6)
West	33 (20.0)
Clinic role	
Clinic leader^c^	17 (10.3)
Clinical staff	142 (86.1)
Other	6 (3.6)
Missing	0
Primary clinic setting^d^	
PTSD specialty care	67 (40.6)
Other mental health	80 (48.5)
PTSD and other mental health	18 (10.9)
Number of patients in the sample, mean (SD)	7.68 (6.30)

Abbreviation: PTSD, posttraumatic stress disorder.

^a^ Defined in administrative data.

^b^ Therapist self-identified race and ethnicity from investigator-defined categories. Race and ethnicity categories with fewer than 7 people were either aggregated or reported as less than 7 to minimize risk of reidentification.

^c^ Titles of leaders included clinic director, assistant director, or team leader.

^d^ Clinic assigned to therapist for 80% or more of their patients during trauma-focused psychotherapy.

Two-thirds (105 [63.6%]) had been treating veterans with PTSD for at least 5 years. Fifty-eight (35.2%) therapists reported burnout (3 or more on the burnout measure). The odds of burnout were elevated for therapists in the South vs the Northeast (odds ratio [OR], 5.39; 95% CI, 1.82-20.02; *P* = .005). Therapists with and without burnout did not differ on any other variable included in Table 1.

### Patient Characteristics and Clinically Meaningful Improvement Distribution

Characteristics of the 1268 patients (961 [75.8%] male) treated by participating therapists are presented in Table 2. The odds of having a therapist with burnout were higher for patients seen in the South compared with the Northeast (OR, 4.95; 95% CI, 3.15-8.13; *P* < .001). Otherwise, there were no apparent differences between the patients of therapists with and without burnout. Four hundred and thirty-one (34%) patients met criterion for clinically meaningful improvement (a reduction of 15 points or more in PCL-5 scores).

**Table 2.** Characteristics of 1268 Patients Who Received Trauma-Focused Psychotherapy for Posttraumatic Stress Disorder

Characteristic	Patients, No (%)
Sex^a^	
Female	307 (24.2)
Male	961 (75.8)
Missing	0
Military service era	
Afghanistan or Iraq	485 (38.2)
Persian Gulf	468 (36.9)
Vietnam	170 (13.4)
Korean	4 (0.3)
Post Vietnam	133 (10.5)
Other	8 (0.6)
Missing	0
Race^a^	
Asian	17 (1.3)
Black	276 (21.8)
Hawaiian or Pacific Islander	20 (1.6)
Multiracial	13 (1.0)
Native American	8 (0.6)
White	853 (67.3)
Missing	81 (6.4)
Ethnicity^a^	
Hispanic	98 (7.7)
Not Hispanic	1118 (88.2)
Missing	52 (4.1)
Current marital status	
Married or partnered	856 (67.5)
Divorced or separated	255 (20.1)
Widowed	13 (1.0)
Never married and single	97 (7.6)
Missing	47 (3.7)
Education	
Less than high school	8 (0.6)
High school	255 (20.1)
Some college or trade school	381 (30.0)
College	198 (15.6)
>College	100 (7.9)
Missing	326 (25.7)
Employment status	
Employed	587 (46.3)
Unemployed	419 (33.0)
Retired	170 (13.4)
Missing	92 (7.3)
Housing stability	
Stable housing	1152 (90.9)
Lack of stable housing	94 (7.4%)
Missing	22 (1.7%)
Census region for therapy	
Midwest	300 (23.7)
Northeast	193 (15.2)
South	503 (39.7)
West	272 (21.5)
Index trauma for therapy	
Combat	668 (52.7)
Other trauma	221 (17.4)
Military sexual trauma	243 (19.2)
Other sexual trauma	46 (3.6)
Multiple sources	52 (4.1)
Missing	38 (3.0)
Multiple trauma history	
Childhood trauma history	718 (56.6)
PTSD service connection	472 (37.2)
Psychiatric comorbidities in prior year	833 (65.7)
Multiple trauma history	718 (56.6)
Childhood trauma history	472 (37.2)
PTSD service connection	833 (65.7)
Psychiatric comorbidities in prior year	
Trauma-related disorders	1248 (98.4)
Depressive disorders	899 (70.9)
Anxiety disorders	560 (44.2)
Alcohol use disorders	254 (20.0)
Other substance-related and addictive disorders	194 (15.3)
Bipolar and related disorders	135 (10.6)
Schizophrenia and other psychotic disorders	18 (1.4)
No. psychiatric comorbidities in prior year, mean (SD)	3.01 (1.34)
Charlson Comorbidity Index, mean (SD)	0.52 (1.09)
Baseline PCL-5, mean (SD)	50.5 (14.0)
Age, mean (SD), y	46.7 (13.9)

Abbreviation: PCL-5, PTSD Checklist for *Diagnostic and Statistical Manual of Mental Disorders* (Fifth Edition).

^a^ Defined in administrative data.

### Therapy Delivery Measures and Clinically Meaningful Improvement

Patient dropout, observed in 587 (46.3%) patients, was associated with reduced odds of clinically meaningful improvement (OR, 0.15; 95% CI, 0.11-0.19; *P* < .001). Therapists’ adherence was not associated with clinically meaningful improvement (OR, 1.09; 95% CI, 0.95-1.25; *P* = .22). Therapists’ session spacing was associated with reduced odds of clinically meaningful improvement such that for every additional 3 days between sessions, the odds of clinically meaningful improvement decreased almost 20% (OR, 0.80; 95% CI, 0.70-0.92; *P* = .002).

### Burnout and Therapy Delivery Measures

Burnout was not associated with patient dropout (OR, 0.98; 95% CI, 0.78-1.24; *P* = .88), therapists’ adherence (OR, 0.95; 95% CI, 0.68-1.34; *P* = .76) or session spacing (OR, 0.99; 95% CI, 0.88-1.10; *P* = .79). Given the apparent lack of dependence on burnout, they did not qualify for consideration as potential mediators.^41^

### Burnout and Clinically Meaningful Improvement

Adjusting for treatment-initiation time period and case-mix, therapists reporting burnout had significantly lower odds of having patients experience clinically meaningful improvement compared with therapists without burnout (Table 3). The odds of clinically meaningful improvement for a patient seen by a therapist who reported burnout were 0.63 (95% CI, 0.48-0.85; *P* = .002) times that for a patient seen by a therapist who did not report burnout, adjusting for covariates. Among the 1268 patients, 120 (28.3%) of the 424 seen by therapists who reported burnout and 311 (36.8%) of the 844 seen by therapists without burnout experienced clinically meaningful improvement in PTSD symptoms.

**Table 3.** Multivariable Logistic Regression Model Evaluating the Relative Odds of Patient Clinically Meaningful Improvement in PTSD Symptoms

Characteristic	OR (95% CI)^a^	*P* Value
Therapist burnout	0.63 (0.48-0.85)	.002
Time period for patient treatment initiation		
Early COVID-19 pandemic	0.84 (0.61-1.16)	.28
During COVID-19 pandemic	0.92 (0.68-1.24)	.58
Pre COVID-19 pandemic	1 [Reference]	NA
Patient characteristics		
PCL-5 scores at baseline	1.37 (1.20-1.55)	<.001
Age, y	1.01 (1.00-1.02)	.33
Retired vs other employment status^b^	1.63 (1.08-2.45)	.02
Lack of stable housing vs other housing status^c^	1.64 (1.04-2.58)	.04
Past year depression diagnosis vs no depression diagnosis	0.68 (0.52-0.88)	.004

Abbreviations: NA, not applicable; OR, odds ratio; PTSD, Posttraumatic Stress Disorder; PCL-5, PTSD Checklist for *Diagnostic and Statistical Manual of Mental Disorders *(*Fifth Edition*).

^a^ Model included random intercept to account for clustering of patients within therapists.

^b^ Reference includes employed, unemployed, and missing.

^c^ Reference includes stable housing and missing.

**Figure 1.**
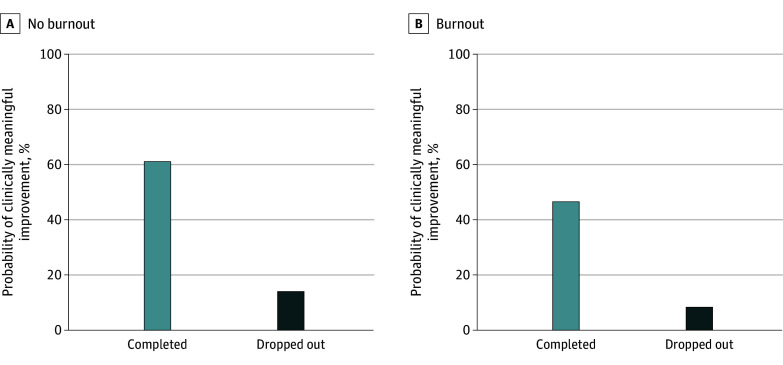
Estimated Probabilities of a Patient Experiencing Clinically Meaningful Improvement Given Therapist Burnout and Patient Dropout Covariates set at their mean or modal values.

**Figure 2.**
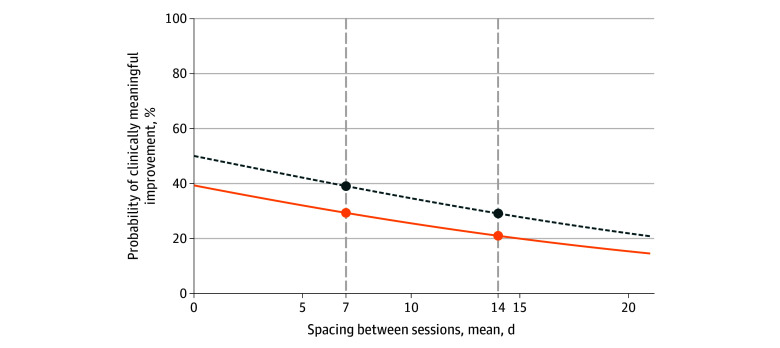
Estimated Probability of Clinically Meaningful Improvement Over Increasing Session Spacing, Stratified by Therapist Burnout Covariates set at their mean or modal values. The solid line indicates burnout, and the dotted line indicates no burnout.

The odds of clinically meaningful improvement given therapist burnout remained lower after we further adjusted for patient dropout (adjusted odds ratio [aOR], 0.56; 95% CI, 0.39-0.79; *P* = .001). Figure 1 displays the model estimated probabilities of a patient experiencing clinically meaningful improvement given therapist burnout and patient dropout. Similarly, the odds of clinically meaningful improvement for patients treated by therapists who reported burnout remained lower when we added session spacing to the case-mix adjusted model (aOR, 0.65, 95% CI, 0.49-0.86; *P* = .003). Figure 2 illustrates the change in the model estimated probability of clinically meaningful improvement over increasing session spacing, stratified by therapist burnout. When we set session spacing to 7 days, there was a 9.7% (95% CI, 3.5%-15.9%; *P* = .002) reduction in the fitted probability of clinically meaningful improvement for patients seen by therapists with burnout compared with therapists without burnout. Set at 14 days, the difference between therapists with and without burnout was 8.1% (95% CI, 2.9%-13.3%; *P* = .002). Changing session spacing from 7 to 14 days, the probability of clinically meaningful improvement decreased 8.3% (95% CI, 1.2%-15.5%; *P* = .02) among therapists with burnout and 10.0% (95% CI, 1.7%-18.2%; *P* = .02) among therapists without burnout.

Sensitivity analyses using the consensus-based definition of clinically meaningful improvement (10 or more point reduction in PCL-5 scores) showed comparable results across unadjusted and adjusted models (eFigure in Supplement 1).

## Discussion

In this prospective cohort study, 35.2% of therapists reported burnout, which was slightly lower than the 38.2% of clinicians who reported burnout on VHA’s 2019 Mental Health Provider Survey.^7^ This is the second study of which we are aware to examine the association between burnout and patient-reported outcomes from psychotherapy and the first to use a prospective design. Consistent with the prior study,^13^ therapists’ self-report of burnout was negatively associated with patients’ probability of benefiting from evidence-based psychotherapy. Among therapists who reported burnout, the odds that patients experienced clinically meaningful improvement in PTSD symptoms were reduced by approximately one-third. The association of burnout may be particularly evident in the context of psychotherapy because psychotherapy requires a high level of clinician interpersonal engagement throughout an episode of care.

Burnout was not associated with TFP effectiveness through patient dropout, therapists’ mean levels of adherence, or session spacing. Future research should examine additional potential explanations for the association between burnout and clinical effectiveness. Therapists who are worn out emotionally may have a reduced capacity to individualize their treatment approach, demonstrate empathy, form a strong working alliance, or facilitate engagement with emotionally charged trauma memories. Therapists with burnout symptoms may be less able to convey expectations for therapeutic benefit, a critical component of treatment success.^43,44^ Alternatively, the observed association between burnout and outcome may result from unmeasured contextual factors in the community or workplace that affect therapists’ well-being and patients’ treatment response. Understanding the reasons for the association between burnout and outcomes in psychotherapy could inform clinician training and interventions to reduce burnout and improve treatment effectiveness.

The need for novel and improved interventions for psychological trauma is well-recognized.^18,21^ However, it is equally important to address modifiable factors that limit the effectiveness of existing evidence-based interventions already in practice. These findings suggest that interventions to reduce therapist burnout might also result in more patients experiencing clinically meaningful PTSD symptom relief from guideline-recommended psychotherapies. Findings further confirm the benefit of providing an adequate dose in terms of the number of and days between sessions.^18,45,46^

How can we best take action to support clinicians and reduce burnout? Low-certainty evidence indicates that interventions to increase compassion, gratitude, or mindfulness may improve resilience of health care professionals.^47^ Yet, management interventions that support workplace control and improve staffing may be preferred over and more effective than those directed toward personal well-being.^48,49^ This is consistent with a view that while burnout manifests in individuals, it is rooted in systems and that system-level interventions to address the causes of occupational burnout are needed.^1,29,30^

### Strengths and Limitations

Strengths of this study include a prospective design, a national sample of therapists and patients, and the use of a patient-reported outcome rather than clinician self-rating of the quality of care. We also examined outcomes across clinicians delivering comparably effective interventions and controlled for patient case-mix.

This study also has limitations. We used a single-item burnout measure that focused on the emotional exhaustion component of burnout^26,27^ and assessed burnout once before the COVID-19 pandemic. We controlled whether each therapy episode was initiated prior to, in the early stages of, or during the COVID-19 pandemic. However, a stronger design would have included repeated assessments of burnout using a more comprehensive, continuous measure and evaluated how changes in burnout were associated with therapy delivery and patient outcomes. While geographic variation in burnout is intriguing, interpretation is limited by heterogeneity within census regions. Unfortunately, there were too few therapists per medical center to examine smaller geographic levels. We also note that the therapist sample was homogeneous regarding race and ethnicity. Finally, factors other than burnout may have accounted for the difference in patient outcomes.

## Conclusions

This prospective cohort study suggests that clinician burnout was negatively associated with patient outcomes from evidence-based psychotherapies. Findings support research to test the hypothesis that interventions to reduce burnout may improve outcomes from guideline-recommended psychotherapies for PTSD. Future work should determine when and how burnout is associated with intervention delivery and patient outcomes.

## Notes

Supplement 1. **eAppendix.** Potential Mediators of the Association Between Burnout and Clinically Meaningful Improvement **eFigure.** Odds Ratios (95% CIs) for the Association Between Clinician Burnout and Patient Clinically Meaningful Improvement

Supplement 2.
Data Sharing Statement
