# Supplementary material for: Clinician Burnout and Effectiveness of Guideline-Recommended Psychotherapies
Source: JAMA Netw Open. 2024 Apr 17;7(4):e246858. doi: 10.1001/jamanetworkopen.2024.6858 (PMC11024738; doi:10.1001/jamanetworkopen.2024.6858)
Supplement: Supplement 1. — eAppendix. Potential Mediators of the Association Between Burnout and Clinically Meaningful Improvement eFigure. Odds Ratios (95% CIs) for the Association Between Clinician Burnout and Patient Clinically Meaningful Improvement [file jamanetwopen-e246858-s001.pdf]

Supplemental Online Content

Sayer NA, Kaplan A, Nelson DB, Wiltsey Stirman D, Rosen CS. Clinician burnout and effectiveness of guideline-recommended psychotherapies. *JAMA Netw Open*. 2024;7(4):e246858. doi:10.1001/jamanetworkopen.2024.6858

eAppendix 1. Potential Mediators of the Association Between Burnout and Clinically Meaningful Improvement
eFigure 1. The OR (95% CIs) for the Association Between Clinician Burnout and Patient Clinically Meaningful Improvement

This supplemental material has been provided by the authors to give readers additional information about their work.

© 2024 Sayer NA et al. *JAMA Network Open.*

eAppendix 1. Potential Mediators of the Association Between Burnout and Clinically Meaningful Improvement

For the study's secondary aim, we sought to explain how or why therapist burnout was associated with the effectiveness of guideline-recommended psychotherapies for posttraumatic stress disorder. We planned to evaluate three therapy delivery factors as potential mediators. Our conceptual model linking the variables is illustrated below.

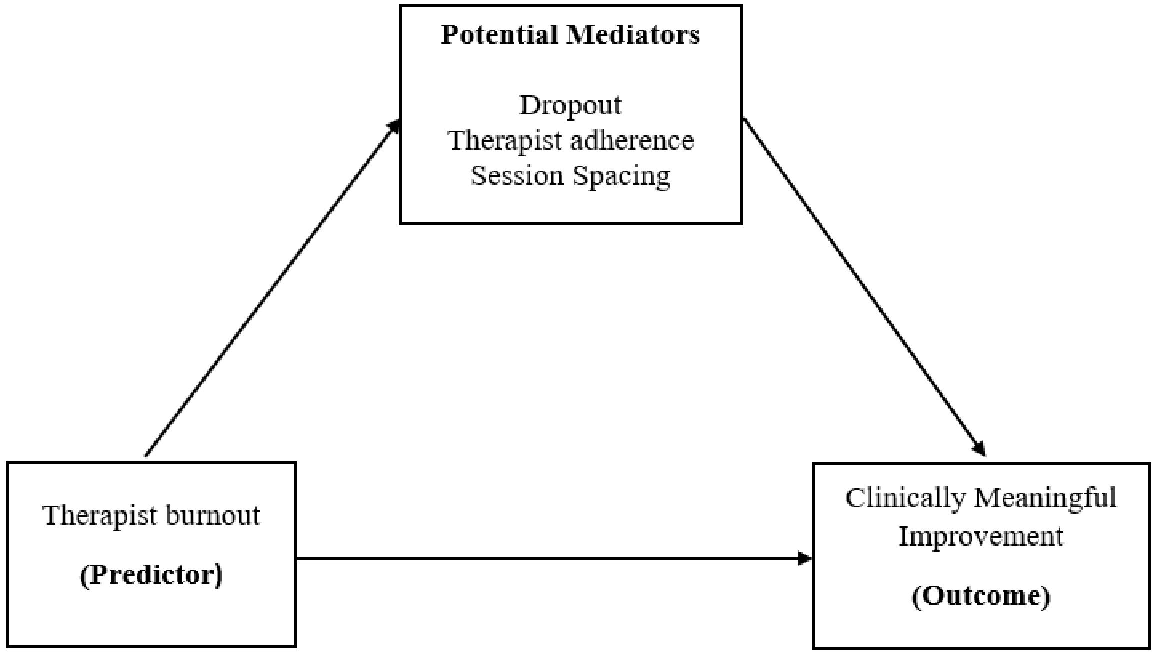

Mediation analysis aims to decompose the total effect of the predictor on the outcome into those contributed by the predictor and those contributed indirectly by the mediators. Potential mediators must meet two eligibility criteria – temporal precedence (the predictor precedes the mediator) and association.[41] Because the predictor (burnout) preceded the therapy delivery measures (patient dropout, therapist adherence, session spacing), the criterion of temporal precedence for the predictor was met. As reported in this article, the criterion of association between the predictor and candidate potential mediators was not met. None of three therapy delivery measures was significantly associated with burnout (*P*s >.76). Therefore, dropout, therapist adherence and session spacing did not qualify as potential mediators and mediation analyses were not implemented.

© 2024 Sayer NA et al. *JAMA Network Open.*

eFigure 1. **The OR (95% CIs) for the Association Between Clinician Burnout and Patient Clinically Meaningful Improvement**

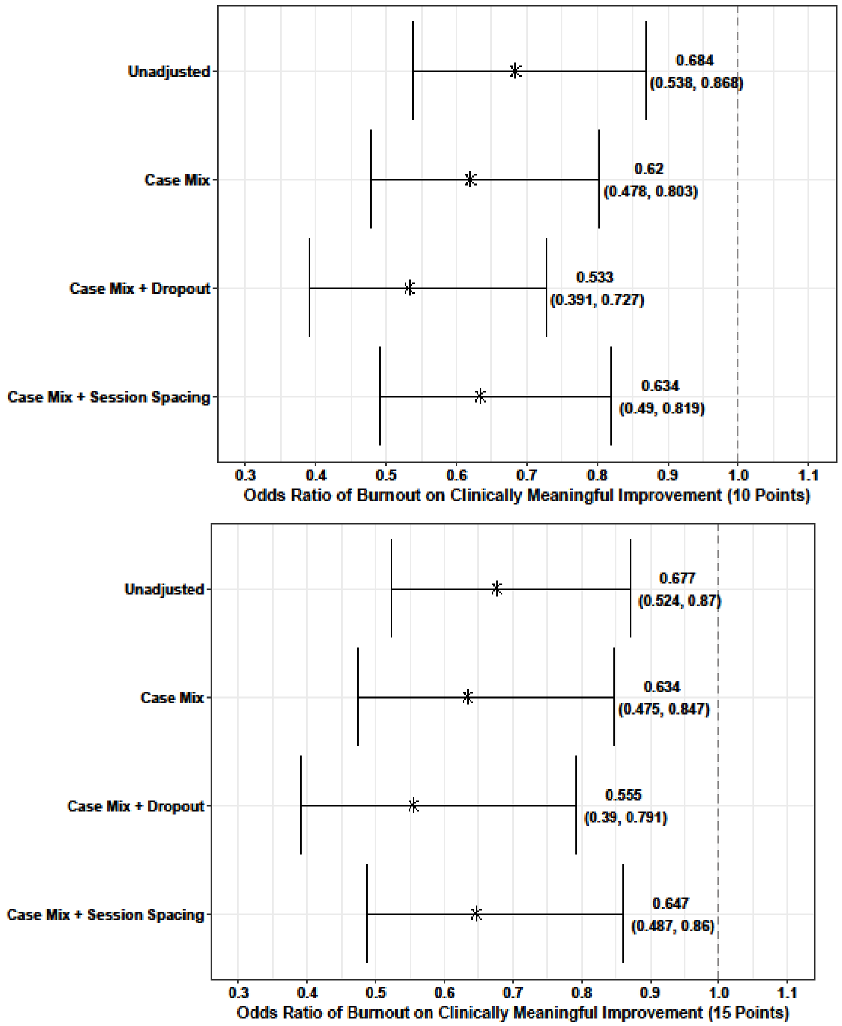

Abbreviations: PCL-5, PTSD Checklist for DSM-5.
Case-mix model adjusted for time period for treatment initiation (pre COVID-19 pandemic, early COVID-19 pandemic, during COVID-19 pandemic) and patient characteristics associated with clinically meaningful improvement at $P < .05$. Patient characteristics included in the model defining clinically meaningful improvement as ≥ 10-point reduction in PCL-5 scores were baseline PTSD symptoms, childhood trauma, retired from employment, census region for therapy, lack of stable housing and past year psychotic disorder diagnosis. Patient characteristics included in the model defining clinically meaningful improvement as ≥ 15-point reduction in PCL-5 scores were baseline PTSD symptoms, age, retired from employment, lack of stable housing and past year depression diagnosis.
[a]Defined as ≥ 10 point and ≥ 15-point reduction in PCL-5 scores

© 2024 Sayer NA et al. *JAMA Network Open.*
