# Supplementary material for: Clinician Burnout and Effectiveness of Guideline-Recommended Psychotherapies
Source: JAMA Netw Open. 2024 Apr 17;7(4):e246858. doi: 10.1001/jamanetworkopen.2024.6858 (PMC11024738; doi:10.1001/jamanetworkopen.2024.6858)
Supplement: Supplement 2. — Data Sharing Statement [file jamanetwopen-e246858-s002.pdf]

Data Sharing Statement

Sayer. Clinician Burnout and Effectiveness of Guideline-Recommended Psychotherapies. *JAMA Netw Open*. Published April 17, 2024. doi:10.1001/jamanetworkopen.2024.6858

Data
Data available: Yes
Data types: Deidentified participant data
How to access data: A de-identified data set is available upon reasonable request from the first author at nina.sayer@va.gov
When available: beginning date: 08-01-2024

Supporting Documents
Document types: None

Additional Information
Who can access the data: researchers whose proposed use of the data has been approved
Types of analyses: for a specified purpose
Mechanisms of data availability: after approval of a proposal and with a signed use agreement
Any additional restrictions: VA Privacy Officer will need to review and approve use of these data for individuals outside of VHA and means of data sharing
